# 1. Yellow Hogs. 2, Verminous Jaundice

**Published:** 1900-08

**Authors:** A. S. Wheeler

**Affiliations:** Biltmore Farms, N. C.


### 1. YELLOW HOGS. 2, VERMINOUS JAUNDICE.

By A. S. Wheeler, V.M.D.,
BILTMORE FARMS, N. C.

1. During the summer months of 1891 and 1892, while acting in the capacity of meat-inspector for the Louisiana State Board of Health, I encountered several shipments of yellow hogs from Tennessee at the Crescent City Slaughter-house. All the tissues were discolored, and especially the adipose tissue. Suspecting jaundice, although the condition of the liver and bile did not bear out the suspicion, I had the chemist of the board make an examination to determine whether or not the discoloration was caused by the bile *salts*. The results of these tests disproved the jaundice theory.

Correspondence with two of the principal shippers did not solve the difficulty, although they both agreed that the cause was in the feeding, but did not agree as to what that feed was.

The odor and taste of these hogs was of a disagreeable fishy character. I regret my inability to explain more definitely the cause of the yellow in these hogs. A simple statement of this condition may bring forth something more complete upon the subject from those who have been in a position to investigate during the past few years, during which such an impetus has been given to meat-inspection.

2. I have met with one case in which the lumen of the bile-duct was completely occluded by an echinorrhynchus gigas. There was a profound jaundice, especially of the fat; the duct was much thickened and indurated; the liver enlarged and dark; gall-bladder contained about two ounces of inspissated bile. This case was seen in the Crescent City Slaughter-house.

Another case similiar to the foregoing occurred in one of the pigs in the Biltmore herd. This pig had been unthrifty for some time, and was put in the cull-lot. Three or four days before his death he had been off feed and dull. His age was about five months.

Post-mortem revealed the same picture as given above; the liver weighed two pounds, the pig weighing thirty-eight pounds. The obstructing worm in this case was an ascaris suilla.

